# Supplementary material for: Identification of Freezing-Responsive microRNAs and Their Targets in Chinese Jujube by Small RNA and Degradome Sequencing
Source: Int J Mol Sci. 2026 Sep 4;27(17):7912. doi: 10.3390/ijms27177912 (PMC13566890; doi:10.3390/ijms27177912)
Supplement: Supplementary file 1 [file ijms-27-07912-s001.zip › Supplementary Material S2/novel_miR_98.pdf]

Provisional ID	:	novel_miR_98
Score total	:	2664.8
Score for star read(s)	:	3.9
Score for read counts	:	2656.8
Score for mfe	:	2.5
Score for randfold	:	1.6
Score for cons. seed	:	
Total read count	:	5223
Mature read count	:	5163
Loop read count	:	33
Star read count	:	27

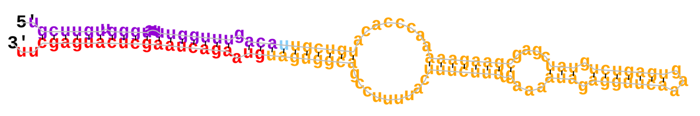

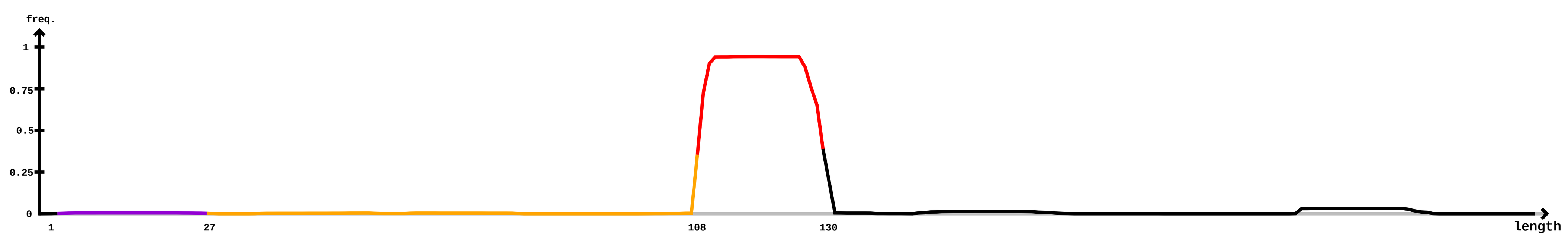

Star **Mature**

```
5'- augcuuguuggguauuuugguuugacauugcuguacacccaaaaagaagcgagcuaugucugagugaaacuuggagauaaaaguuuucuuucauuuuccgacggugauguaagacuaagcucaugagcuuauuauccuaggucgaaacaaguugauaggauccccuuuuuugggucccсaugucccucccauguggugacguggggcauaaaaaaggaaagagagggaugguguuucucucgcuuuuag -3' obs
    augcuuguuggguauuuugguuugacauugcuguacacccaaaaagaagcgagcuaugucugagugaaacuuggagauaaaaguuuucuuucauuuuccgacggugauguaagacuaagcucaugagcuuauuauccuaggucgaaacaaguugauaggauccccuuuuuugggucccсaugucccucccauguggugacguggggcauaaaaaaggaaagagagggaugguguuucucucgcuuuuag     exp
    ..((((((.((((...(((((((.((((((((((........((((((((....(((.(((((((...))))))).)))...).))))))).........))))))))).))))))))))))))))).....(((((..(((((......))))))))))..((((((((.((((((((((((........)).))))))))))).)))))))))((((.((((((.......)))))).))))...   reads  mm  sample
```

read	reads	mm	sample
augcuuguuggguCuuuugguuu	1	1	JCK-1
augcuuguuggguauuNugguuugaca	1	1	LT-2
augcuuguuggguCuuuugguuugaca	1	1	JCK-1
.ugcuuguuggguauuuugguA	1	1	LT-3
.ugcuuguuggguCuuuugguuugac	1	1	JT-3
.ugcuuguuggguCuuuugguuugaca	1	1	JT-2
.ugcuuguuggguauuuugguuugCca	1	1	JCK-1
.ugcuuguuggguauuuuggCuugaca	1	1	LT-3
.ugcuuguuggguCuuuugguuugaca	1	1	LCK-3
..gcuuguuggguauuuugguA	1	1	LT-1
..gNuuguuggguauuuugguu	1	1	JT-1
..Ucuuguuggguauuuugguuugaca	1	1	LT-3
..gcuuguuggguauuuugguuuUaca	1	1	JT-3
..gcuuguuggguauuuugguuugacC	1	1	LT-2
...cuuguuggguCuuuugguu	1	1	LT-2
...cuuguuggguCuuuugguuug	1	1	LT-2
...cuuguuggguCuuuugguuugac	1	1	LCK-3
...cuuguuggguauuuugguuugCc	1	1	LT-1
...cuuguuggguCuuuugguuugac	1	1	LT-2
...cCuguuggguauuuugguuugaca	1	1	LT-1
....uuguuggguauuuuggGuug	3	1	LT-2
....uuguuggguauuuCgguuuga	1	1	LT-3
....uuguuggguauuuugUuuugac	1	1	LT-2
....uuguuggguauuuugguuugacC	1	1	LT-1
........uggguauuuugguuugCca	1	1	JCK-2
..................................acacccaaaaagaagcgagc	1	0	LT-3
..................................acacccaaaaagaagcgagc	1	0	LT-1
...................................cacccaaaaagaagcgagc	3	0	LCK-3
...................................cacccaaaaagaagcgagc	1	0	LT-3
...................................cacccaaaaagaagcgagcu	4	0	LT-1
....................................acccaaaaagaagcgagc	2	0	LT-3
....................................acccaaaaagaagcgagcua	1	0	LT-3
......................................ccaaaaagaagcgagcua	1	0	LT-3

Star Mature

augcuuguugggguauuuugguuugacauugcuguacacccaaaaagaagcgagcuaugucugagugaaacuuggagauaaaaguuuucuuucauuuuccgacggugauguaagacuaagcucaugagcuuauuauccuaggucgaaacaaguugauaggaucccccuuuuuugggucccccaugucccucccauguggugacgugggggcauaaaaaaggaaagagagggaugguguuucucucgcuuuuag

...aagaagcgagcuaAgucugagu...	1	1	LCK-2
...agcgagcuaugucugagugaaacuuggaga...	1	0	LCK-1
...cgagcuaugucugagugaaacuug...	1	0	LT-3
...gagcuaCgucugagugaaacuuggagaua...	2	1	LT-2
...gcuaCgucugagugaaacuuggagaua...	2	1	LT-2
...ugagugaaacuuggagau...	8	0	LCK-3
...ugagugaaacuuggagau...	1	0	LT-2
...gagugaaacuuggagaua...	3	0	LT-1
...gacggugaCguaagacuaagcuc...	1	1	LT-2
...acggugaCguaagacuaagcucaugagcuu...	1	1	JCK-2
...cggugaCguaagacuaagc...	1	1	LT-2
...cggugaCguaagacuaagcucauga...	1	1	LCK-3
...gugaCguaagacuaagcuc...	1	1	LT-2
...ugaCguaagacuaagcucauga...	1	1	LT-2
...ugaCguaagacuaagcucaugagcuu...	2	1	LT-2
...gaCguaagacuaagcucaugag...	1	1	LT-3
...aNguaagacuaagcucauga...	1	1	JCK-1
...aCguaagacuaagcucaugag...	1	1	LCK-2
...aCguaagacuaagcucaugagc...	1	1	JCK-1
...aCguaagacuaagcucaugagcu...	2	1	LT-2
...aNguaagacuaagcucaugagcu...	1	1	LT-3
...Cguaagacuaagcucaugagcuu...	1	1	LT-3
...guCagacuaagcucauga...	1	1	LT-2
...guaagacuaagcucauga...	12	0	JT-1
...guaagacuaagcucauga...	35	0	JT-2
...guaagacuaagcucauga...	80	0	LCK-2
...guaagacuaagcucauga...	29	0	LT-2
...guaagacuaagcucauga...	47	0	JT-3
...guaagacuaagcucauga...	1	0	JCK-1
...guaagacuaagcucauga...	22	0	LCK-3
...guNagacuaagcucauga...	1	1	LT-2
...guaagacuaagcucauga...	4	0	JCK-3
...guaagacuaagcucauga...	15	0	LT-1
...guaagacuaagcucauga...	2	0	JCK-2
...guaagacuaagcucauga...	71	0	LCK-1
...guaagacuaagcucauga...	15	0	LT-3
...Uuaagacuaagcucauga...	2	1	LT-2
...Auaagacuaagcucauga...	1	1	LCK-2
...guaagacuaagcucaugag...	26	0	LCK-3
...guaagacuaagcucaugag...	39	0	LT-2
...guaagacuaagcucaugag...	15	0	LCK-2
...guaagGcuaagcucaugag...	1	1	JCK-2
...guaagacuaagcucaugag...	33	0	LT-3
...guCagacuaagcucaugag...	2	1	LT-2
...guaagacuaagcucaugag...	22	0	JT-2
...guaagacuaagcucaugag...	4	0	JCK-2
...guaagacuaagcucaugag...	5	0	JCK-1
...guaagacuaagcucaugag...	3	0	JCK-3
...guaagacuaagcucaugag...	14	0	LCK-1
...guaagacuaagcucaugag...	18	0	LT-1
...guaagacuaagcucNugag...	1	1	LCK-1
...guaagacuaagcucaugag...	32	0	JT-3
...guaagacuaagcucaugagc...	25	0	LT-2
...guaagacuaagcucaugagc...	9	0	JCK-1
...guaagacuaagcucaugagc...	27	0	LT-3
...guaagacuaagcucaugagc...	22	0	LT-1
...guaagacuaagcucaugagc...	13	0	JCK-3
...guaagacuaagcucaugagc...	10	0	LCK-3
...guaagacuaagcucaugagc...	7	0	JT-3
...guaagacuaagcucaugagc...	7	0	JT-2
...guaagacuaagcucaugagc...	20	0	JCK-2
...guaagacuaagcucaugagcu...	45	0	LCK-3
...guaagacuaagcucaugagcu...	15	0	JCK-3
...guaagacuaagcucaugagAu...	1	1	JCK-3
...guaagacuaagcucaugagcu...	70	0	LT-3
...guaagacuaagcucaugagcu...	72	0	LCK-1
...Uuaagacuaagcucaugagcu...	1	1	LT-3
...guaagacuaagUucaugagcu...	1	1	JT-3
...guaagacuaagcucaugagcu...	55	0	LT-1
...guaagacuaagcucaugagcu...	13	0	JCK-2

Star **Mature**

augcuuguugggguauuuugguuugacauugcuguacacccaaaaagaagcgagcuaugucugagugaaacuuggagauaaaaguuuucuuucauuuuccgacggugauguaagacuaagcucaugagcuuauuauccuaggucgaaacaaguugauaggauccccuuuuuugggucccccaugucccucccauguggugacgugggggcauaaaaaaggaaagagagggaugguguuucucucgcuuuuag

guaagacuaagcucaugagcu	69	0	LCK-2
guaagacuaagcucaugagcu	9	0	JT-3
guaagacuaagcucaugagcu	13	0	JCK-1
guaagacuaagcucaugagcA	1	1	JCK-3
guaagacuaagcucaugagcu	8	0	JT-2
guaagacuaagcucaugagcu	72	0	LT-2
guaagacuaagcucaugagcC	1	1	LT-3
guCagacuaagcucaugagcuu	1	1	JT-1
guCagacuaagcucaugagcuu	1	1	JT-3
guaagacuaagUucaugagcuu	1	1	LT-1
Auaagacuaagcucaugagcuu	1	1	LCK-1
guaagacuaagcucaugagcuu	101	0	LCK-1
guaagacuaagcucaugagcuu	28	0	JCK-2
guaagacuaagcucCugagcuu	1	1	LCK-2
guCagacuaagcucaugagcuu	1	1	LT-2
guaagacuaagcucaugagcuu	37	0	JT-2
guaagacuaagcucaugagcuu	39	0	JT-1
guaagacuaagUucaugagcuu	1	1	JT-2
guaagacuaagcucaugagcuu	64	0	LCK-3
guaagacuaagcucaugagcuu	23	0	JCK-3
guaagacuaagcucaugagcuu	66	0	JT-3
guaagacuaagcucaugagcuu	84	0	LT-1
guaagacuaagcucaugCgcuu	1	1	LT-3
guaagacuaagcucaugagcuu	84	0	LCK-2
guCagacuaagcucaugagcuu	1	1	LCK-2
guaagacuaagcucaugagcuu	123	0	LT-2
guaagacuaagcucaugagcuu	18	0	JCK-1
guaagacuaagcucaugagcuu	108	0	LT-3
guaagacuaagcucaugagcuuauu	3	0	LCK-3
guaagacuaagcucaugagcuuauuauccu	2	0	LCK-3
guaagacuaagcucaugagcuuauuauccu	1	0	LT-3
guaagacuaagcucaugagcuuauuauccu	2	0	JCK-1
guaagacuaagcucaugagcuuauuauccu	3	0	JCK-2
uaagacuaagcucaugag	6	0	JCK-2
uaagacuaagcucaugag	58	0	LCK-2
uaagacuaagcucaugag	22	0	LT-1
uaagacuaaUcucaugag	1	1	LCK-2
uaagacuaagcucaugag	25	0	JT-2
uaagacuaagcucaugag	3	0	JCK-3
uaagacuaagcucaugag	5	0	JT-1
uaagacuaagcucaugag	8	0	JCK-1
uaagacuaagcucaugag	120	0	LT-2
uaagacuaagcucauCag	1	1	LT-2
uaagacuaagcucaugag	45	0	LCK-3
uaagacuaagcucaugag	83	0	LCK-1
uaagacuaagcucaugag	38	0	JT-3
uaagacuaagcucaugag	41	0	LT-3
uaagacuaCgcucaugag	1	1	LCK-1
uaagCcuaagcucaugagc	1	1	LT-2
uaagacuaagcucaugagc	36	0	JCK-2
uaagacuaagcucaugagc	17	0	JCK-1
uaagacuaagcucaugagc	28	0	LT-2
uaagacuaagcucaugagc	17	0	JT-3
uaagacuaagcucaugagc	46	0	LT-1
uaagacuaagcucaugagc	24	0	JCK-3
uaagacuaagcucaugagc	14	0	LCK-3
uaagacuaagcucaugagc	45	0	LT-3
Caagacuaagcucaugagc	1	1	JCK-1
uaagacuaagcucaugagc	11	0	JT-1
uaaUacuaagcucaugagc	1	1	LT-1
uaagacuaagcucaugagc	11	0	JT-2
uaagCcuaagcucaugagcu	1	1	LT-2
uaagacuaagUucaugagcu	1	1	LT-2
uaagCcuaagcucaugagcu	1	1	JCK-3
uaagacuaagcucaugagcC	1	1	LCK-3
uaagacuaagcucaugagcu	63	0	LCK-3
uaagacuaagcucaugagcu	5	0	JT-3
uaagacuaagcucaugagcu	16	0	JCK-3
uaagacuaagcucaugagcu	105	0	LT-2
uaagacuaagcucaugagcu	18	0	JT-1

Star **Mature**

augcuuguuggguauuuugguuugacauugcuguacacccaaaaagaagcgagcuaugucugagugaaacuuggagauaaaaguuuucuuucauuuuccgacggugauguaagacuaagcucaugagcuuauuauccuaggucgaaacaaguugauaggaucccccuuuuuugggucccccaugucccucccauguggugacgugggggcauaaaaaaaggaaagagagagggauggugguuucucucgcuuuuag

uaaAacuaagcucaugagcu	1	1	JCK-3
uaagacuaagcucaugagcu	76	0	LT-3
uaagacuaagcucaugagcu	24	0	JCK-2
uaagacuaagcucaugagcA	1	1	LCK-3
uaagacuaagcucaugagcA	1	1	JCK-2
uaagacuCagcucaugagcu	1	1	LT-1
uaagacuaagcucaugagcu	8	0	JT-2
uaagacuaagcucaugagcu	12	0	JCK-1
uaagacuaagcucCugagcu	1	1	LCK-3
uaagacuaagcucaugagcu	89	0	LT-1
uaagacuaagcucaugagcuu	48	0	JCK-1
Caagacuaagcucaugagcuu	1	1	LT-1
uaagacuGagcucaugagcuu	1	1	JCK-3
uaagacuaagcucaugagcuu	52	0	JCK-2
uaagacuaagcucaugagcuu	84	0	LCK-1
uaagacuaagcucaugagcuu	119	0	LT-3
uaagacuaagcucaugagcuu	107	0	LCK-3
uaagacuaagcucaugagcuu	41	0	JCK-3
uaagacuaagcucaugagcuu	75	0	JT-3
uaagacuaagcucaugagAuu	1	1	LCK-2
uaagacuaagAucaugagcuu	1	1	LCK-2
uaagacuaagcucaugagcuu	39	0	JT-2
uaagacuaagUucaugagcuu	1	1	JT-3
uaagacuaagcucaugagcuu	99	0	LT-1
uaagacuaagcucaugaAcuu	1	1	LCK-2
uaagacuaagcucaugagcuu	56	0	LCK-2
uaagacuCagcucaugagcuu	1	1	LT-2
uaagacuaagcucaugagcuu	98	0	LT-2
uCagacuaagcucaugagcuu	1	1	LCK-2
uaagacuaagcucaugagcuu	81	0	JT-1
uaCgacuaagcucaugagcuu	1	1	JT-1
uaagacuaagcucaNgagcuu	1	1	LCK-1
uaagacuaagcucaugagcuua	1	0	LT-3
uaagacuaagcucaugagcuua	4	0	LT-1
uaagacuaagcucaugagcuuauu	2	0	JCK-2
uaagacuaagcucaugagcuuauu	1	0	LT-3
aagacuaagcucaugagc	23	0	JCK-1
aagacuaagcucaugagc	31	0	JCK-3
aagacuaagcucaugagc	42	0	JCK-2
aagacuaagcucaugCgc	1	1	LT-1
aCgacuaagcucaugagc	1	1	LT-3
aagacuaagcucCugagc	1	1	JCK-1
aagacuaagcucaugagc	25	0	LT-1
aagacuaagcucaugagc	32	0	LT-3
aagacuaagcucaugagc	17	0	LCK-3
aagacuaagcucaugagc	12	0	LT-2
aagacuaagcucaugagc	7	0	JT-3
aagacuaagcucaugagcu	10	0	JCK-1
aagacuaagcucaugagcu	24	0	JCK-2
aagacuaagcucaugagcu	76	0	LT-3
aagacuaagcucaugagcu	25	0	JT-2
aagacuaagcucauUagcu	1	1	LT-1
aagacuaagcucCugagcu	1	1	JCK-3
aagacuaagcucaugagcA	1	1	LT-2
aagacuaagcucaugagcu	30	0	JT-1
aagacuaagcucaugagcu	26	0	JT-3
aagacuaagcuNaugagcu	1	1	JT-1
Uagacuaagcucaugagcu	1	1	LT-3
aagacuaagcucaugagcA	1	1	LCK-3
aagacuCagcucaugagcu	1	1	LT-2
aagacuaCgcucaugagcu	1	1	LT-1
aagacuaagcucaugagcu	99	0	LCK-3
aagacuaagcucaugagcu	70	0	LT-1
aagacuaagcucaugagcu	42	0	LT-2
aagacuaagcucaugagcu	17	0	JCK-3
aagacuaagcucaugagcuu	20	0	LCK-1
aagacuaagcucaugagcuu	69	0	LT-2
aagacuaagcucaugagcuu	16	0	JCK-1
aagacuaaUcucaugagcuu	5	1	LT-2
aagacuaagcucaugagcuu	33	0	LCK-2

Star **Mature**

augcuuguuggguauuuugguuugacauugcuguacacccaaaaagaagcgagcuaugucugagugaaacuuggagauaaaaguuuucuuucauuuuccgacggugauguaagacuaagcucaugagcuuauuauccuaggucgaaacaaguugauaggauccccuuuuuuggguccccaugucccucccauguggugacgugggggcauaaaaaaggaaagagagggaugguguuucucucgcuuuuag

aagacuaagcucaugagcuu	12	0	JCK-3
aagacuaagcucaugagcuu	3	0	JT-1
aagacuaagcucaugagcuu	11	0	JT-3
aagacuaagcucaugagcuu	64	0	LCK-3
aagacuaagcucCugagcuu	1	1	JT-3
aagacuaagUucaugagcuu	1	1	JCK-2
aagacuaagcucaugagcuu	53	0	LT-3
aagacuaagcucaugagcuu	31	0	LT-1
aagacuaagcucaugagcuu	10	0	JT-2
aagacuCagcucaugagcuu	1	1	LT-3
aagacuaagcucaugagcuu	11	0	JCK-2
aagacuaagcucaugaUcuuauuaucc	1	1	JT-3
agacuaagcucaugagcu	11	0	LCK-3
agacuaagcucaugagcu	7	0	JT-2
agacuaagcucaugagcu	2	0	JCK-3
agacuaagcucaugagcu	2	0	JCK-1
agacuaagcucaugagcu	21	0	JT-3
agacuaagcucaugagcu	15	0	LT-1
agacuaagcucaugGgcu	1	1	JCK-1
agacuaagcucaugagcA	1	1	LT-3
agacuaCgcucaugagcu	1	1	LT-2
agacuaagcucaugagcu	36	0	LT-3
agacuaagcucaugagcu	4	0	JCK-2
agacuaagcucaugagcA	1	1	LT-1
agacuaagcucaugagcu	39	0	LT-2
agacuaagcucaugagcu	5	0	JT-1
agacuaagcucaugagcuu	1	0	JCK-1
agacuaagcucaugagcuA	1	1	LT-3
agacuaagcucaugagcuA	1	1	LT-1
agacuaagcucaugagcuu	4	0	LT-1
agacuaagcucaugagcuu	10	0	LT-3
agacuaagcucaugagcuu	9	0	JT-3
agacuaagcucaugagcuA	3	1	LT-2
agacuaagUucaugagcuu	1	1	JT-2
agacuaagcucaugagcuu	4	0	LCK-3
agacuaagcucaugagcuu	2	0	JCK-2
agacuaagcucaugagcuA	3	1	LCK-3
agacuaagcucaugagcuu	7	0	LT-2
agacuaagcucaugagcuu	17	0	JT-1
agacuaagcucaugagcuu	6	0	JT-2
agacuaagcucaugagcuuauuauccu	1	0	JCK-2
gacuaagcucaugagcuu	2	0	LT-3
gacuaagcucaugagcuuauuauccu	1	0	LT-3
cuaagcucaugagcuuaG	2	1	JT-2
cuaagcucaugagcuuauuauccu	1	0	JCK-3
cuaagcucaugagcuuauuauccu	2	0	JCK-2
cuaagcucaugagcuuauuauccuaggucg	1	0	LT-2
uaagcucaugagcuuauuauccuagguc	1	0	JCK-2
agNucaugagcuuauuauccuagguc	1	1	LT-2
auNagcuuauuauccuag	1	1	LT-2
auccuaggucgCaacaaguuga	1	1	LT-3
aacaaguugauaggaucc	1	0	LT-1
aacaaguugauaggaucc	4	0	LT-2
aacaaguugauaggauccc	3	0	LT-3
aacaaguugauaggauccc	2	0	LT-1
aacaaguugauaggaucccc	1	0	LT-2
aacaaguugauaggaucccc	1	0	LT-3
aacaaguugauaggaucccc	2	0	JT-2
aacaaguugauaggaucccc	5	0	JT-3
aacaaguugauaggauccccuuu	3	0	LT-1
aacaaguugauaggauccccuuu	2	0	LT-3
acaaguugauaggauccc	1	0	LT-3
acaaguugauaggauccc	1	0	LT-1
acaaguugauaggaucccc	2	0	LT-1
acaaguugauaggauccccu	1	0	LT-1
acaaguugauaggauccccu	1	0	JCK-2
acaaguugauaggauccccuu	1	0	LT-1
acaaguugauaggauccccuuu	1	0	LT-1
acaaguugauaggauccccuuuu	1	0	LT-3
caaguugauaggaucccc	2	0	LT-3

Star **Mature**

augcuuguuggguauuuuugguuugacauugcuguacacccaaaaagaagcgagcuaugucugagugaaacuuggagauaaaaguuuucuuucauuuuccgacggugauguaagacuaagcucaugagcuuauuauccuaggucgaaacaaguugauaggaucccccuuuuuugggucccc augucccucccauguggugacguggggg cauaaaaaaggaaagagagggaugguguuucucucgcuuuuag

caaguugauaAgaucccc	1	1	LT-1
caaguugauaggaucccc	1	0	LT-2
caaguugauaggaucccccu	2	0	LT-3
caaguugauaggaucccccu	4	0	LT-1
caaguugauaggaucccccuu	1	0	LT-3
caaguugauaggaucccccuu	1	0	LT-1
caaguugauaggaucccccuuu	3	0	LCK-3
caaguugauaggaucccccuuu	3	0	LT-1
caaguugauaggaucccccuuuuu	2	0	LT-3
caaguugauaggaucccccuuuuuu	3	0	LCK-3
aaguugauaggaucccccuuu	1	0	LT-3
aaguugauaggaucccccuuuuu	1	0	LT-1
aguugauaggaucccccuuu	2	0	LT-3
aguugauaggaucccccuuu	5	0	LT-2
aguugauaggaucccccuuuu	1	0	LT-1
aguugauaggaucccccuuuu	3	0	LCK-3
guugauaggaucccccuuu	2	0	LT-3
guugauaggaucccccuuuu	1	0	LT-3
guugauaggaucccccuuuuuu	1	0	LT-1
uugauaggaucccccuuuu	1	0	LT-1
uugauaggaucccccuuuu	1	0	LT-3
uugauaggaucccccuuuuu	2	0	LT-3
cccuuuuuuCgguccccaugucccc	1	1	LCK-3
aAaaaaaaggaaagagagggau	4	1	LT-2
uaaaaaaaggaaagagagg	5	0	JT-3
uaaaaaaaggaaagagagg	7	0	LT-1
uaaaaCaggaaagagagg	1	1	JT-2
uaaaaaaCgaaagagagg	2	1	LT-3
uaaaaaaaggaaagagagg	1	0	JCK-3
uaaaaaaaggaaagagagg	1	0	JCK-2
uaaaaaaaggaaagagagg	4	0	LCK-3
uaaaaaaaggaaagagagg	7	0	JT-2
uaaaaaaaggaaagagagg	2	0	LT-3
uaaaaaaaggaaagagaggg	10	0	JT-2
uaaaaaaaggaaagagaggg	11	0	JT-3
uCaaaaaaggaaagagaggg	1	1	LT-1
uaCaaaaaggaaagagaggg	1	1	LT-2
uaaaaaaaggaaagagaggg	16	0	JT-1
uaaaaaaaggaaagagaggg	3	0	LT-2
uaaaaaaaggaaagagaggg	6	0	LT-1
uaaaaaaaggaaagagaggg	3	0	LT-3
uaaaaaaaggaaagagaggga	12	0	JT-3
Aaaaaaaaggaaagagaggga	1	1	LT-3
uaaaaaaaggaaagagaggga	2	0	LT-3
uaaaaaaaggaaagagaggga	3	0	JCK-2
uaaaaaaaggaaagagaggga	2	0	LCK-3
uaaaaaaaggaaagagaggga	10	0	JT-2
uaaaaaaaggaaagagaggga	2	0	LT-1
uaaaaaaaggaaagagagggau	1	0	LT-2
uaaaaaaaggaaagagagggau	6	0	LT-3
uaaaaaaaggaaagagagggaug	10	0	LT-1
uaaaaaaaggaaagagagggaug	2	0	LT-2
uaaaaaaaggaaagagagggaug	5	0	LT-3
uaaaaaaaggaaagagagggaug	1	0	JCK-2
uaaaaaaaggaaagagagggaug	22	0	JT-1
uaaaaaaaggaaagagagggaugg	1	0	JT-2
uaaaaaaaggaaagagagggauNg	1	1	JT-2
uaaaaaaaggaaagagagggaugg	1	0	JT-3
aCaaaaggaaagagagggaug	1	1	LT-1
aaaaaggaGagagaggga	1	1	LT-2
aaCaaggaaagagagggaug	1	1	JT-2
aaaCaggaaagagagggaugg	1	1	JT-1
aaaagAaaagagagggaugg	1	1	LT-1
